# Neutralization of interleukin-17A alleviates burn-induced intestinal barrier disruption via reducing pro-inflammatory cytokines in a mouse model

**DOI:** 10.1186/s41038-019-0177-9

**Published:** 2019-12-18

**Authors:** Yajun Song, Yang Li, Ya Xiao, Wengang Hu, Xu Wang, Pei Wang, Xiaorong Zhang, Jiacai Yang, Yong Huang, Weifeng He, Chibing Huang

**Affiliations:** 1 Department of Urology, Xinqiao Hospital, the Third Military Medical University, No.83 Xinqiao Street, Shapingba District, Chongqing, 400038 China; 2 Institute of Burn Research, Southwest Hospital, State Key Laboratory of Trauma, Burns and Combined Injury, the Third Military Medical University, No.30 Gaotanyan Street, Shapingba District, Chongqing, 400038 China

**Keywords:** IL-17A, Burn, Intestinal mucosa barrier, Pro-inflammatory cytokines, Vγ4^+^ T cells, Interleukin-17A

## Abstract

**Background:**

The intestinal barrier integrity can be disrupted due to burn injury, which is responsible for local and systemic inflammatory responses. Anti-inflammation strategy is one of the proposed therapeutic approaches to control inflammatory cascade at an early stage. Interleukin-17A (IL-17A) plays a critical role in inflammatory diseases. However, the role of IL-17A in the progression of burn-induced intestinal inflammation is poorly understood. In this study, we aimed to investigate the effect of IL-17A and associated pro-inflammatory cytokines that were deeply involved in the pathogenesis of burn-induced intestinal inflammatory injury, and furthermore, we sought to determine the early source of IL-17A in the intestine.

**Methods:**

Mouse burn model was successfully established with infliction of 30% total body surface area scald burn. The histopathological manifestation, intestinal permeability, zonula occludens-1 expression, pro-inflammatory cytokines were determined with or without IL-17A-neutralization. Flow cytometry was used to detect the major source of IL-17A^+^ cells in the intestine.

**Results:**

Burn caused intestinal barrier damage, increase of intestinal permeability, alteration of zonula occludens-1 expressions, elevation of IL-17A, IL-6, IL-1β and tumor necrosis factor-α (TNF-α), whereas IL-17A neutralization dramatically alleviated burn-induced intestinal barrier disruption, maintained zonula occludens-1 expression, and noticeably, inhibited pro-inflammatory cytokines elevation. In addition, we observed that the proportion of intestinal IL-17A^+^Vγ4^+^ T subtype cells (but not IL-17A^+^Vγ1^+^ T subtype cells) were increased in burn group, and neutralization of IL-17A suppressed this increase.

**Conclusions:**

The main original findings of this study are intestinal mucosa barrier is disrupted after burn through affecting the expression of pro-inflammatory cytokines, and a protective role of IL-17A neutralization for intestinal mucosa barrier is determined. Furthermore, Vγ4^+^ T cells are identified as the major early producers of IL-17A that orchestrate an inflammatory response in the burn model. These data suggest that IL-17A blockage may provide a unique target for therapeutic intervention to treat intestinal insult after burn.

## Backgroud

Burn injury is among the most common forms of trauma. Inflammation caused by burn injury in lungs, livers, kidneys, and intestines has been observed and studied [1–3]. This immediate systemic inflammatory response that spreads throughout the whole body leads to sepsis, multiple organ dysfunction and other complications, bringing about 75% of all deaths in patients [4].

Notably, following severe burn injury, the intestine is inevitably subjected to ischemia resulting from mesenteric vasoconstriction, subsequently, reperfusion of blood occurs, exacerbating fluctuations of oxygen levels [5]. The ischemia/reperfusion injury initiates a local inflammatory response in the gut with activation of resident immune cells, such as T helper type 1 (Th1), Th17, and γδT cells [6–8], which generate pro-inflammatory cytokines like interleukin (IL)-17A, IL-6,IL-1β, tumor necrosis factor-α (TNF-α), et al. [9, 10]. The release of these pro-inflammatory cytokines can be directly or indirectly damaging to intestinal epithelial cells. Massive epithelial cell death, combined with apoptosis and loss of intercellular tight junction proteins would lead to a breakdown of the epithelial barrier, increasing intestinal permeability and bacterial translocation [11]. Therefore, efforts have been made to define the consequence of burn in terms of lymphocyte cytokine identity, and multiple studies have demonstrated that there is an early and quick onset of inflammatory response symptom characterized by production of certain pro-inflammatory cytokines in the intestine [12].

If the pro-inflammatory state is not well regulated or controlled, chemotactic and cytotoxic factors in the gut promote immune cell infiltration, further contributing to the development of the systemic inflammatory response. Therefore, therapeutic strategies aimed at preventing pro-inflammatory response at an early stage would provide an opportunity to limit burn-induced intestinal barrier damage.

As one of the most important pro-inflammatory cytokines, IL-17A has been found to be highly involved in several models of inflammatory or autoimmune diseases [13], such as colitis, asthma, multiple sclerosis, rheumatoid arthritis and renal allograft rejection. As one of the identified members of IL-17 family, IL-17A induces many other pro-inflammatory mediators such as IL-6, IL-23 by T cells [14], and stimulates the production of IL-1β and TNF-α by macrophages [15]. It mediates inflammation and immune response by recruiting neutrophils and regulating other inflammatory cells [16], propagating a broad immuno-inflammatory response. Studies have shown increased IL-17-expressing macrophages in active inflammatory bowel disease [17], and Finnerty and his colleagues demonstrated that circulating IL-17 levels significantly increased early after burn injury in pediatric patients, as well as in a mouse burn model [18]. These initial findings indicate that IL-17 may be deeply involved in the intestinal inflammatory response after burn injury.

On the other hand, it is reported that anti-IL-17A antibody treatment on mice with hypersensitivity pneumonitis resulted in milder inflammation [19]. Neutralization of IL-17A improved prognosis of severe septic mice [20], delayed progression of silica-induced lung inflammation [21], rescued amyloid-β-induced neuro-inflammation [22]. Anti-IL-17A monoclonal antibody has been applied successfully in clinical trials for the treatment of inflammatory diseases recently [23, 24]. Their success is suggestive that in a burn model, application of IL-17A neutralization to modulate IL-17A-mediated inflammation is entirely plausible.

To date, the role of IL-17A in burn-induced intestinal barrier disruption has been seldom investigated. In the present study, we firstly constructed the burn model, and inhibited IL-17A activity via the application of anti-IL-17A monoclonal antibody. Then, the intestinal barrier damage and levels of pro-inflammatory cytokines were studied to explicate correlations between them. Thereafter, an attempt was made to further identify the major source of IL-17A. This study opens the possibility of mitigating burn-induced intestinal damage in the early stage, thereby providing a novel potential target of burn therapy.

## Methods

### Animals

A total of 15 C57BL/6 female mices (divided into three groups, sham group, burn group and burn+anti-IL-17A antibody group, *n* = 5 in each group) weighing 20-25 g were purchased from the Animal Center of the Third Military Medical University (Chongqing, China), and were housed in a specific pathogen-free environment with free access to water and food. They were acclimatized for 1 week prior to the experiment in a temperature-controlled room (24 ± 1 °C) with a 12-h light/dark cycle. All animal studies were approved by the Animal Care and Use Committee of the Third Military Medical University, and all experimental protocols were approved by the Medical and Ethics Committee of Xinqiao Hospital, the Third Military Medical University, Chongqing, China.

### Burn injury procedure

The burn model was established according to the method previously used in our laboratory [11]. Briefly, mice were anesthetized via intraperitoneal (i.p.) injection of ketamine/xylazine. After shaving and fixing dorsal surface, mice were immersed in 90 °C water for 10 s with an area of 30% total body surface area, to produce a full-thickness burn; the sham group was immersed in water set to 37 °C. Following burn or sham procedures, 1 ml Ringer’s solution was immediately administered i.p. for resuscitation. After mice were fully awake, they were returned to the animal facility. This method is proved successful in establishing a third-degree burn model in both our and other previous studies. Mice were sacrificed 24 h following burn injury for further study.

### IL-17A neutralization

Mice in the burn+anti-IL-17A antibody group received intraperitoneal injection of 100 μg anti-mouse IL-17A antibody (clone eBioMM17F3, eBioscience, San Diego, CA, USA) one day before and at the time of burn injury induction, while 100 μg isotype antibody (BioLegend, San Diego, CA, USA) in saline was injected into the burn group in the same way at the same time.

### Intestinal permeability measurement *in vivo*

After being anesthetized, the mice were laparotomized through a midline incision 12 h after burn or sham treatment. A 5-cm segment of jejunum, which began at 3 cm distal to the Treitz ligament, was dissociated and tied by suture at both of the lateral ends. Fluorescein isothiocyanate (FITC) -labeled dextran (200 μl,10 mg/ml) was injected into the jejunal segment. One hr. later, blood specimens were taken from inferior vena cava, followed by centrifugation at 2000 g for 10 min at 4 °C. The serum was collected for fluorescence intensity detection at an excitation wavelength of 480 nm and emission wavelength of 520 nm (Varioskan Flash, Thermo), then the intestinal permeability was calculated according to a standard curve of reference FITC-labeled dextran.

### Histopathological and immunofluorescent examination of the distal ileum

Mice were sacrificed 24 h after burn injury, the distal ileum was harvested, flushed, and immersed in 10% buffered formalin for 1 day for fixation. After dehydration in ethanol, all specimens were embedded in paraffin, sectioned at a thickness of 5 μm, and stained with hematoxylin and eosin (H&E). Histopathological evaluations were performed and scored in a blinded fashion . The distribution of tight junction protein zonula occludens-1 (ZO-1) was assessed with an immunofluorescent assay which was carried out as previously described [11]. In brief, a section of distal ileum was stained with monoclonal rabbit anti-ZO-1 (Invitrogen, Carlsbad, CA) with a dilution of 1:400 in 5% goat serum in phosphate buffered saline (PBS) at 4 °C overnight, and then incubated at a secondary antibody of goat anti-rabbit Alexa-488 IgG (Invitrogen, Carlsbad, CA). Finally, the section was treated with diamidine-2-penylindole (DAPI, Sigma) at the concentration of 1:1000 for 1 h under room temperature, and rinsed with PBS, then covered with a cover slip. Microscopy (BX53, Olympus, Japan) was used to detect the fluorescence.

### Determination of inflammatory cytokines in serum and ileum

To determine the levels of inflammatory cytokines in different groups, blood sample and ileal segments were collected at the time when animals were sacrificed (24 h after burn). For the blood sample taken from inferior vena cava, it was centrifugated at 2000 g for 10 min at 4 °C, then, the serum concentrations of IL-17A, IL-6, IL-1β and TNF-α were measured using murine enzyme-linked immunosorbent assay (ELISA) kits (Dakewe Biotech Corporation, Shenzhen, China) according to the manufacturer’s instructions, and were normalized for the amount of cytokines per mg of total protein in the sample. For the ileal segment, cytokines were assessed by Western-blot. Tissues were flushed, the ileal mucosa was collected, and lysis buffer (KeyGEN, China) was added. Equal amounts of total protein (30 μg) were loaded onto a 12% gel for sodium dodecyl sulfate-polyacrylamide gel electrophoresis (SDS-PAGE), and then the proteins were transferred onto polyvinylidene difluoride (PVDF) membrane (Millipore, Bedford, MA, USA) in the transfer buffer. After the membranes were blocked for 2 h with skim milk in PBS (5% wt/v), they were incubated with antibodies (diluted at 1:500) specific for IL-17A (PA5–79470), IL-6 (M620), IL-1β (M421B) and TNF-α (MM350D) overnight at 4 °C, and β-actin (diluted at 1:2000,MA5–15739) was used as loading control. After being washed with Tris-buffered saline with Tween-20 (TBST), the membranes were incubated with HRP-labeled secondary antibodies (diluted at1:3000, KPL, America) at room temperature for 1 h, and then washed for 3 times with TBST. At last, the chemiluminescence detection kit (Sungene, China) was applied to react with the secondary antibody. The protein bounds were visualized by the Chemi Dox XRS Western blot detection system, and analyzed by Quantity One Image software (Bio-Rad, USA).

### Intraepithelial lymphocyte isolation

Intraepithelial lymphocytes (IELs) were isolated as previously described [25]. Briefly, mouse was sacrificed, its ileal segment was excised and flushed of fecal material in a 100 mm tissue culture dish containing supplemented RPMI-1640, then Peyer’s patches were removed. The ileal segment was opened longitudinally, cut into 0.5 cm strips. The strips were placed into a new 50 mL tube containing 35 mL calcium and magnesium free HBSS (H1040, Solarbio) with 2 mM EDTA and 4 mM dithiothreitol (DTT), and fully digested on a rotary shaker for 1 h at 37 °C. After that, the tissue slurry was passed successively through a mesh filter for 3 times to remove undigested tissue pieces. Subsequently, the cell suspension was put into two a 50 mL tube and centrifuged at 400 g for 5 min at 37 °C. Cells were pelleted and washed by RPMI-1640 for 2 times before being mixed in 5 mL of 40% isotonic Percoll and overlaid onto 5 ml of 70% isotonic Percoll in a 15 mL centrifuge tube. The gradients were centrifuged at 400 g, 30 min, at 4 °C. IELs were collected from the 40/70% cloudy interface area, and washed by centrifugation in RPMI-1640.

### Flow cytometry analysis

IELs were identified by flow cytometry. Blank control (unstained cells) was set up to adjust the voltage so that the intensity of auto-fluorescence was suppressed, to some extent, excluding false positive results. Cells were incubated with Fc receptor (FcR) blocking antibodies (antiCD16/CD32; eBioscience) for 15 min on ice to block nonspecific staining. For surface staining, cells were incubated with anti-CD3 mAb (ab5690, Abcam, UK), anti-TCRγδ mAb (60104PE, STEMCELL), anti-TCRVγ1 mAb (555,321, BD, USA), and anti-TCRVγ4 mAb (552,143, BD, USA) for 30 min at room temperature. Subsequently, intracellular staining with IL-17A mAb (45–7177-82, Thermofisher, USA) or corresponding isotype control Abs (eBioscience) was conducted after fixing and permeabilizing cells according to the manufacturer ‘s instructions (Fix and Perm Cell Permeabilization kit, BD Biosciences, USA). All the samples were detected using an Attune acoustic focusing cytometer (Applied Biosysterm, USA), and analyzed by FlowJo software, (USA).

### Statistical analysis

Data of pathology scores were analyzed by Mann-Whitney test and presented as median with range. Data of OD value, percentage of cells, and cytokine expression were analyzed by t-test or analysis of variance (ANOVA) and are presented as the mean ± standard error of the mean (SEM). A *p* value< 0.05 was regarded as statistically significant. Statistical analysis was performed with SPSS20.0 software (IBM). Graphs were plotted with GraphPad Prism 7.0 software (GraphPad Software).

## Results

### Severe burn caused pathological intestinal changes, increased intestinal permeability, and altered tight junction protein ZO-1 expression

**Fig. 1:**
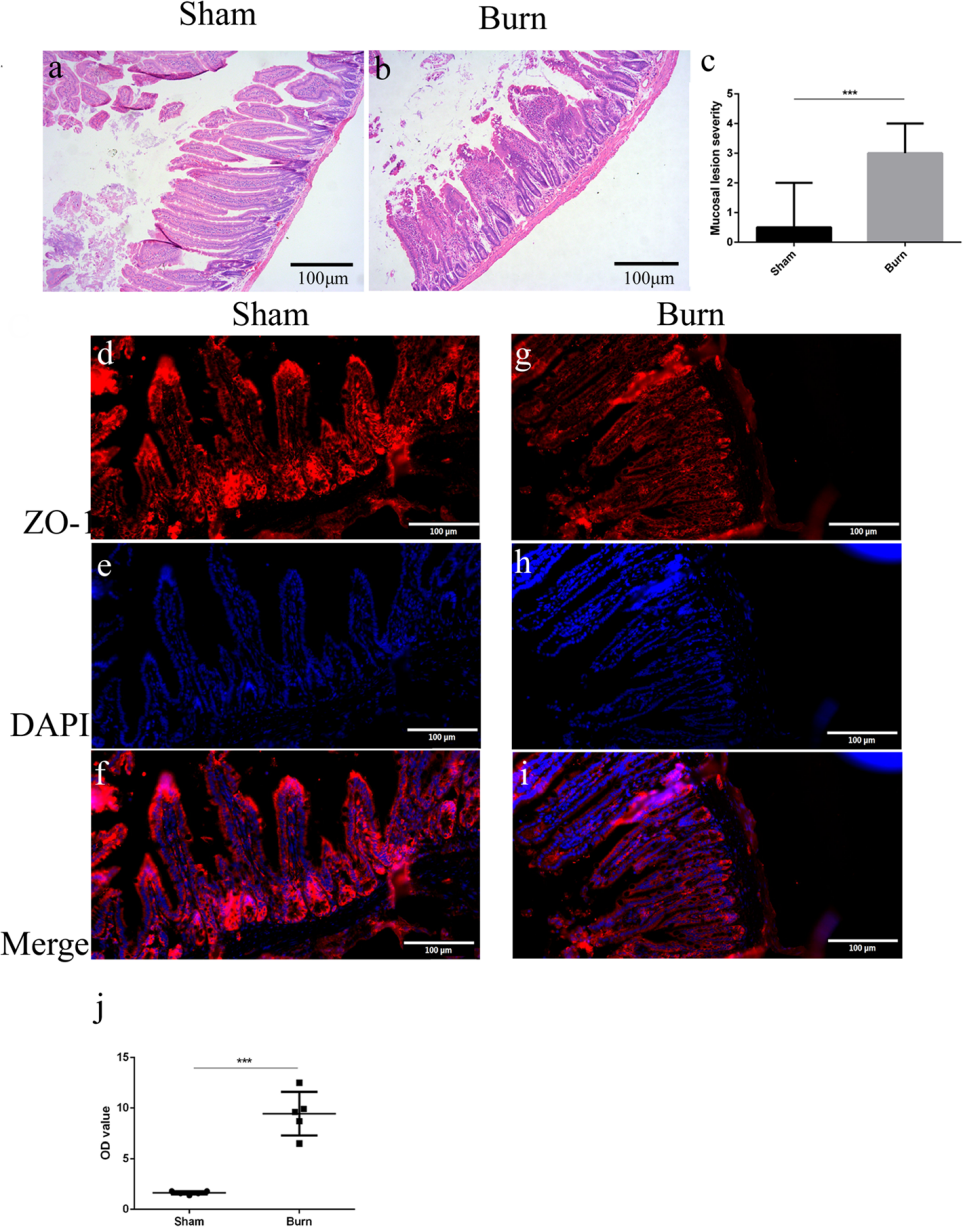
Microscopic appearance of intestine barrier disruption and zonula occludens-1 (ZO-1) alteration induced by burn injury. Ileal sections were stained with hematoxylin and eosin (H&E) (**a**, **b**, 200x), and mucosa lensions were scored in both groups (**c**). Representative immunofluorescent images depicting membrane localization of ZO-1 in sham group (**d**-**f**), and burn group (**g**-**i**), red staining indicates ZO-1, and blue staining indicates nuclei (400x). Intestinal permeability was measured by fluorescein isothiocyanate (FITC)-dextran levels and is expressed as optical density (OD) value (mean ± standard error of the mean (SEM)) (**j**). *n* = 5 per group.***p < 0.001. *DAPI* 4′,6-diamidino-2-phenylindole

Burn-induced pathological intestinal injury is characterized by findings such as destruction of epithelial cells, mucosal thickening, edema, and  infiltration of inflammatory cells. As shown in Fig. 1, there was minimal intestinal lesioning observed in sham group (Fig. 1a), whereas shorter and wider villi and an elevation in intestinal epithelial cells destruction was observed in burn group (Fig. 1b). According to the criteria of Chiu, the pathological findings were evaluated and scored by a single pathologist who was blinded to all the experimental design. When compared with the sham group (0.5, 2), burn group (3, 3) significantly had more pronounced intestinal injury (Fig. 1c). Tight junction proteins have been proven to be critical as structural proteins for the maintenance of mucosa barrier function. To illuminate burn-induced intestinal barrier deficiency, immunofluorescent was applied to evaluate the alteration of tight junction protein expression of ZO-1. Exposure-matched fluorescent intensity was correlated with ZO-1 expression after immunostaining. In the sham group, ZO-1 was detected as red staining and in nearly a line at the most apical compartment of cell-cell junctions (Fig. 1d). In contrast, ZO-1 morphology was more disrupted, and its fluorescence intensity was lower in the burn group (Fig. 1g). The ZO-1 alteration illustrated that a severe burn can impair intestinal barrier integrity.

To further confirm the contribution of burn to intestinal permeability, FITC-dextran levels were measured as described above. FITC-labeled dextran levels in serum in burn group were significantly higher than that of sham group (9.44 ± 0.97 vs 1.64 ± 0.07, *p* < 0.05), as shown in Fig.1j, suggesting that burn injury increased intestinal permeability.

All these data illustrated that severe burn can impair intestinal mucosa barrier, and indicated the successful establishment of a burn-induced intestinal damage model.

### Severe burn-induced intestinal inflammatory cell infiltration, elevated pro-inflammatory cytokine expression in ileum and serum

**Fig. 2:**
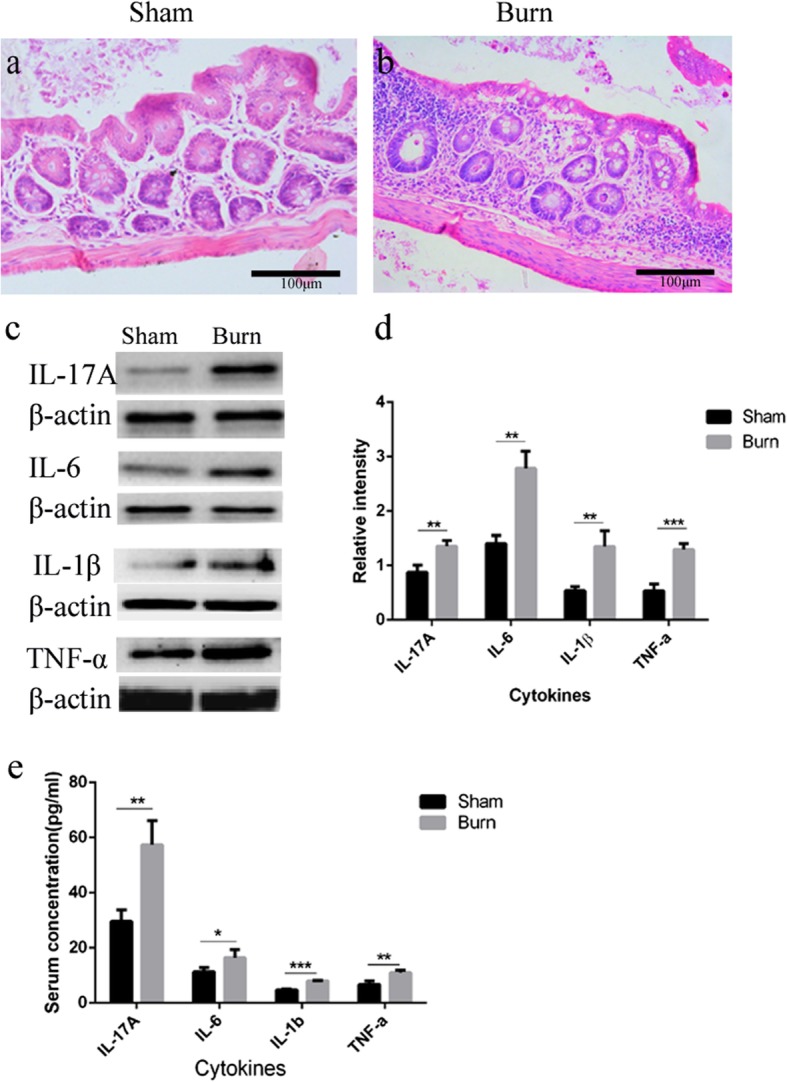
Epithelial inflammatory cell infiltration, and elevated pro-inflammatory cytokine expression induced by burn injury. Ileal sections were stained with hematoxylin and eosin (H&E), and extensive microscopic inflammatory cell infiltration in burn group compared with the sham group was observed (**a**, **b**, 200x). Cytokine protein levels in ileum of both groups were determined by Western blotting (**c**), and a summary of cytokines blots were presented as the ratio of cytokines:β-actin densities (**d**). Cytokine protein levels in serum of both groups were determined by enzyme-linked immunosorbent assay (ELISA), and normalized to pg/ml of total serum volume (**e**). *n* = 5 per group. *p < 0.05, **p < 0.01, ***p < 0.001. *IL* Interleukin, *TNF-α* Tumor necrosis factor-α

H&E staining showed extensive microscopic inflammatory cell infiltration in burn group compared with the sham group (Fig. 2a,b). To determine the involvement of pro-inflammatory cytokines 24 hr after burn, the expression of IL-17A, IL-6, IL-1β, and TNF-α were analyzed both in ileal mucosa by Western blotting, and in serum by ELISA. As shown in Fig. 2(c,d) compared with in the sham group, expression of IL-17A, IL-6,IL-1β, and TNF-α in ileal mucosa was significantly increased in the burn group. Meanwhile, in serum, levels of IL-17A, IL-6,IL-1β, and TNF-α were significantly higher in the burn group than the sham group (Fig. 2e).

These results suggested that severe burn can promote local inflammation in the intestinal mucosa, and ulteriorly contribute to systemic inflammation at an early stage.f

### Neutralization of IL-17A attenuated intestinal pathological changes and ZO-1 alteration, suppressed elevation of intestinal permeability, and inhibited increase of pro-inflammatory cytokine expression

**Fig. 3:**
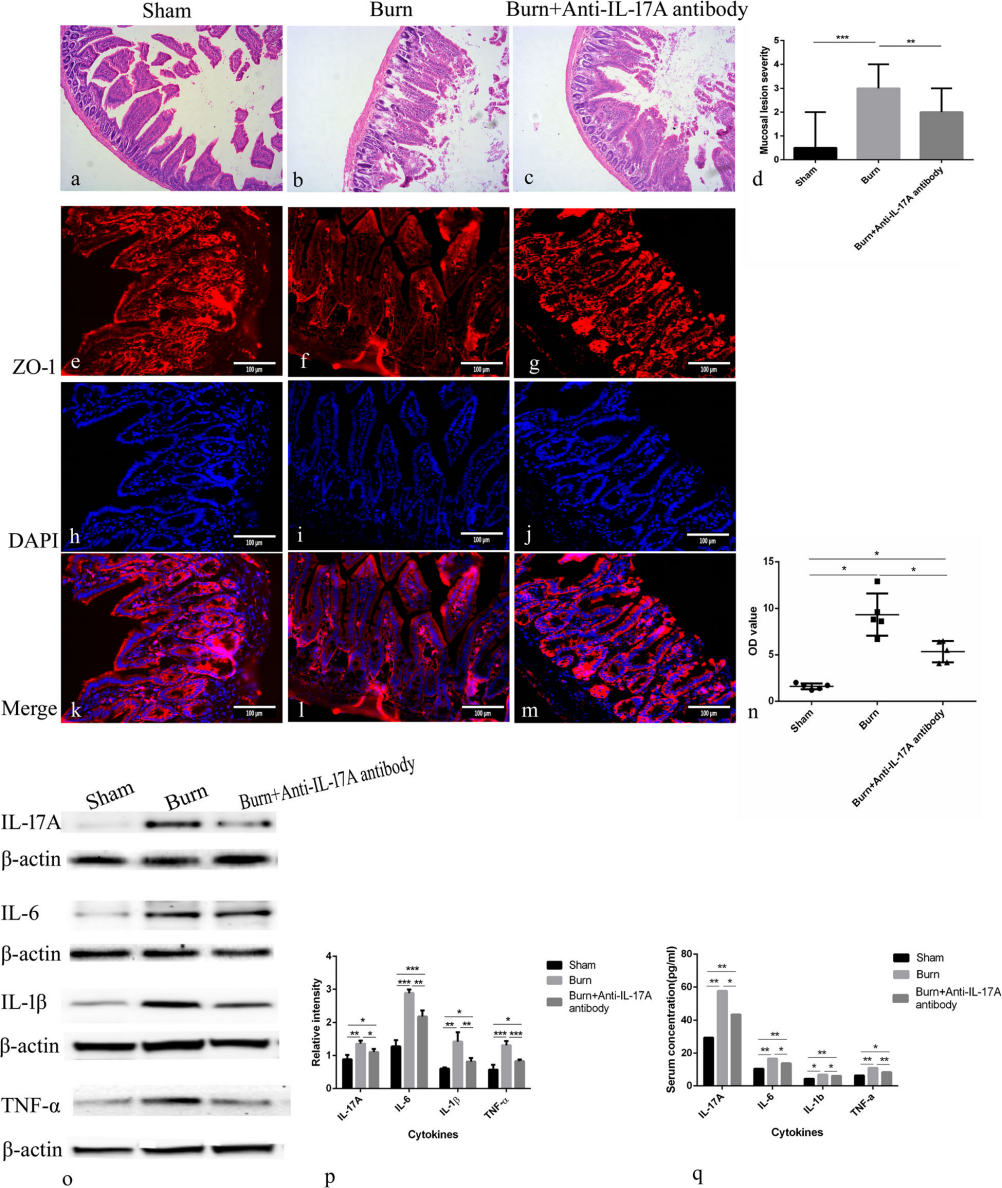
Interleukin (IL)-17A neutralization attenuated intestinal pathological changes and zonula occludens-1 (ZO-1) alteration, suppressed intestinal permeability elevation, and inhibited increases of cytokines. Ileal sections were stained with hematoxylin and eosin (H&E) (**a**, **b**, **c** 200x), and mucosa lensions were scored in each group(**d**). Representative immunofluorescent images depicting membrane localization of ZO-1 in sham group (**e**-**g**), burn group (**h**-**j**), and burn+anti-IL-17A antibody group (**l**-**m**), red staining indicates ZO-1, and blue staining indicates nuclei (400x). Intestinal permeability was measured by fluorescein isothiocyanate (FITC)-dextran levels and is expressed as optical density (OD) value (mean ± standard error of the mean (SEM)) in per group (**n**). Cytokine protein levels in ileum of each group were determined by Western blotting (**o**), and summary of cytokines blots is presented as the ratio of cytokines: β-actin densities (**p**). Cytokine protein levels in serum of each group were determined by enzyme-linked immunosorbent assay (ELISA), and normalized to pg/ml of total serum volume (**q**). *n* = 5 per group. * *p*< 0.05, **p < 0.01, *** *p*< 0.001. *DAPI* 4′,6-diamidino-2-phenylindole

IL-17A is a crucial mediator of inflammatory responses. Previous work from our laboratory demonstrated that epidermal IL-17A production is increased after skin injury, and the administration of IL-17A neutralizing antibody on wound beds significantly improved wound healing. Therefore, in our burn model, IL-17A has been blocked to further investigate its role in burn-induced intestinal damage, and the effects of IL-17A neutralization are depicted in Fig. 3. Intestinal microscopic appearance of the three groups (Sham, Burn, Burn+anti-IL-17A antibody) is shown in Fig.3(a,b,c). Consistent with previous observations, burn group showed overt damaged intestinal mucosa, whereas burn+anti-IL-17A antibody group showed ameliorated intestinal damage. Compared with the sham group (0.5, 2), burn group (3, 3) had more severe intestinal injury, while neutralization of IL-17A attenuated burn-induced pathological changes in the intestine (2, 2) (Fig. 3d).

To assess the effects of anti-IL-17A antibody on the expression of ZO-1, immunofluorescence was performed. Burn group exhibited low expression of ZO-1 at the cell periphery compared to sham group, while burn+anti-IL-17A antibody group showed a similar ZO-1 expression pattern with the sham group (Fig. 3 e-m), which indicated the loss of ZO-1 was attenuated following treatment with anti-IL-17A antibody. FITC-dextran level (Fig.3n) in serum in burn group (1.60 ± 0.31) was significantly higher than that of sham group (9.32 ± 2.27), while the anti-IL-17A antibody group showed decreased FITC-dextran serum levels (5.34 ± 1.16) compared with those of the burn group, indicating blockage of IL-17A suppressed elevation of intestinal permeability.

The Western blotting results (Fig.3o,p) showed that ileal mucosa protein expression of IL-17A, IL-6, IL-1β, and TNF-α in both burn and anti-IL-17A antibody group was significantly greater than that in sham group. Compared with the burn group, the expression of ileal mucosa proteins were significantly down-regulated in the anti-IL-17A antibody group. Serum pro-inflammatory cytokine expression was determined by ELISA which showed increased levels of IL-17A, IL-6, IL-1β, and TNF-α after burn, which were significantly decreased by administration of IL-17A antibody (Fig.3q). These results indicated that neutralization of IL-17A suppresses the increase of the pro-inflammatory cytokine expression.

### Presence of Vγ4^+^ T cells was the major source of early IL-17A production after burn

**Fig. 4:**
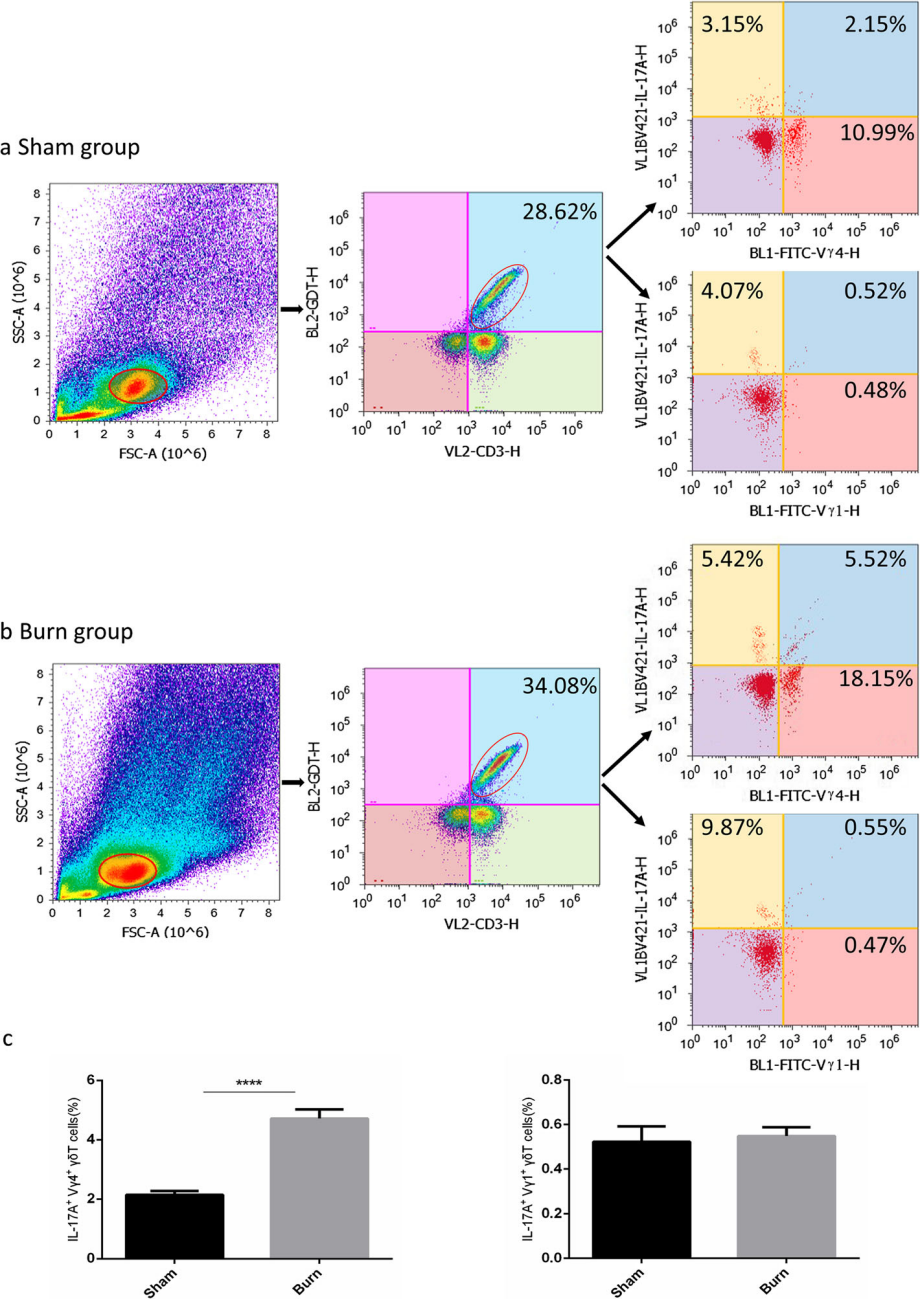
Flow cytometry analysis of interleukin (IL)-17A expression in γδT cells isolated from intestinal epithelial lymphocytes (IELs). IEL cells were gated in the oval, and membrane staining with anti-mouse CD3, TCRγδ, Vγ4^+^ or Vγ1^+^ antibody and intracellular staining with anti-mouse IL-17A antibody were performed. Representative flow cytometric data in each group: sham group (**a**), burn group (**b**). A comparison of percentage between IL-17A^+^Vγ4^+^T cells (left) and IL-17A^+^Vγ1^+^T cells (right) in each group (**c**). The results are presented as mean ± standard error of the mean (SEM), *n* = 5 per group. *** *p*< 0.001

Figure 4 shows that percentage of γδT cells significantly increased in the burn group (b) compared with the sham group (a) (28.62 ± 2.20% vs 34.08 ± 2.41%, *p* < 0.05), while the percentage of IL-17A^+^Vγ4^+^T cells significantly increased in burn group compared with the sham group (4.72 ± 0.36% vs 2.15 ± 0.25%, *p* < 0.05). Few IL-17A^+^Vγ1^+^T cells were detected in sham group and burn group, and there was no significant difference between two groups (0.52 ± 0.06% vs 0.55 ± 0.05%, *p* > 0.05). The above results demonstrated that Vγ4^+^T cells are the main γδT cell subtype secreting IL-17A at the early stage of inflammation in the intestine. However, there were other IL-17A-producing cell subtypes which were unidentified in this experiment and needs further confirmation.

## Discussion

The main original findings of this study are that the intestinal mucosa barrier is disrupted after burn through affecting the expression of pro-inflammatory cytokine, and a protective role of IL-17A neutralization for intestinal mucosa barrier is determined. Furthermore, Vγ4^+^T cell subtype, rather than Vγ1^+^T cell subtype is the dominant source of early IL-17A after burn.

It is well known that IL-17A is an important pro-inflammatory cytokine which prompts macrophages, T cells and epithelial cells to produce and release other chemokines or cytokines to mediate tissue infiltration, inflammation, destruction and remodeling [15, 16]. Thus, IL-17A exerts various functions, and has been implicated in several disease models [26–28]. Previous work from our laboratory demonstrated epidermal IL-17A production is increased after skin injury, and the administration of IL-17A neutralizing antibody on wound beds significantly improved wound healing [29]. Based on that, we evaluated the levels of IL-17A, IL-6, IL-1β and TNF-α in each group and found them significantly increased in burn group, but such up-regulation was markedly inhibited via anti-IL-17A antibody treatment, which may be associated with amelioration of burn-induced damage to the intestine barrier, because IL-6, IL-1β not only directly disrupt intestinal barrier, but also promote the induction of naive T lymphocytes towards IL-17A-producing cells to aggravate inflammatory damage to the intestine. Hence, the results clearly reflected the value of IL-17A blockage in maintaining the integrity of the intestinal mucosal barrier.

We observed de-localized, lower ZO-1 expression early after burn and considered it as a morphology marker of intestinal epithelial injury. Upon the administration of IL-17A antibody, the expression of ZO-1 was stronger than the burn group, which indicated that this treatment contributed to protection of tight junctions after burn, and the suppressed elevation of intestinal permeability further proved this point. Notably, whether IL-17A has pathogenic and/or protectiveeffects on tight junctions is controversial. Function of IL-17A on tight junctions depends on the experiment setting and its interaction with other associated components within the inflammatory context. Jacob S. Lee et al. provided evidence that the IL-17A dependent regulation of the tight junction protein occludin during epithelial injury limits excessive permeability and maintains barrier integrity [30], while Mohammed T. reported that IL-17A rapidly increases the permeability of the small intestinal epithelial barrier by modifying tight junctions including ZO-1 and occludin [31].

To our knowledge, TNF-α has been shown to disrupt tight junctions and increase epithelial barrier permeability in Caco-2 cells [32], and according to Wang L et al., TNF-α stimulation can increase the expression of miR-191a, resulting in the decline of ZO-1 mRNA and protein levels[33]. In the present study, level of TNF-α was up-regulated by burn, but down-regulated by IL-17A antibody. Hence, it is reasonable to speculate that the protective effect of IL-17A neutralization on ZO-1 was partially due to down-regulation of TNF-α in our experiment setting.

To further identify the underlying mechanism of intestinal IL-17A function after burn, the possible source of IL-17A needed to be determined. It is generally accepted that IL-17A is mainly derived from monocyte-macrophage lineages and several subtypes of T lymphocytes such as Th17 cells and γδT cells during different stages of immune response [34–36]. Under certain circumstances, γδT cells are evidenced to be the primary source of early IL-17A production, and display unique functional properties that place them in between adaptive and innate immunity [37, 38]. However, there is little information regarding the phenotypic and functional characteristics of IL-17A-producing γδT cells. It is reported that IL-17A^+^γδT cells secrete IL-17A during the early immune response. According to Kensuke, γδT cells, especially those expressing Vδ1^+^, are the major source of the early IL-17A production [39]. Li da Sun et al. reported Vγ4^+^ T cells are an IL-17A-producing γδT subset during the early phase of chlamydial airway infection in mice [40]. In our previous study, Vγ4^+^T cells provided an early source of IL-17A in a mouse skin transplantation model and accelerated allograft rejection reaction [41]. In mice, IL-17A^+^γδT cells are mainly restricted to the subtypes of Vγ1^+^, Vγ4^+^ and Vγ6^+^ γδT cells [42], and Vγ6^+^T cells are the major γδ T cell sub-population in reproductive tract [43], but not in gastrointestinal tract. On these grounds, we focused on the subtypes of Vγ4^+^ T cells and Vγ1^+^ T cells in the intestine to verify the major source of IL-17A in our burn model. Representative flow cytometry results showed that dominant IL-17A staining was detectedin Vγ4^+^ T cells, but not in Vγ1^+^ T cells. Consistent with our data, Vγ4^+^ T cells comprise the major subtype that produces IL-17A in different experiment models studied previously. We also observed an increase of the percentage of Vγ4^+^ T cells in the after burn, whereas neutralization of IL-17A significantly down regulated the increase.However, the possible mechanism needs to be further investigated.

Importantly, in addition to γδT cells, a variety of cell types, such as innate lymphoid cell 3 (ILC3 cells) [44], NK cells, and Th17 cells, are also able to secrete IL-17A. Other members of the IL17 family are expressed by epithelial cells, including those in the gut, to function as a key factor in the early intestinal immune or inflammatory response. IL17 produced by many cellular sources has wide-ranging effects and downstream transcriptional consequences, some of which are just beginning to be understood.

There are some limitations to our study. First, the number of experimental animals was not large in each group although we could report significance. Second, treatment with IL-17A antibody may also impact other inflammatory cytokines and cell populations, but we did not obtain significant results. Third, we only used one anti-IL-17A antibody concentration, dose, and set one postburn timepoint to examine; optimally, additional experimental perturbations would be studied.

## Conclusions

The main original findings of this study are that the intestinal mucosa barrier is disrupted after burn through affecting the expression of pro-inflammatory cytokines, and a protective role of IL-17A neutralization for intestinal mucosa barrier is determined. Furthermore, Vγ4^+^ T cells were identified as the major early producers of IL-17A that orchestrate an inflammatory response in the burn model. These data suggest that IL-17A blockage may provide a unique target for therapeutic intervention to treat intestinal insult after burn.

## Data Availability

All data generated and/or analyzed during the current study are included in this published article.

## Abbreviations

DAPI: Diamidine-2-penylindole
FITC: Fluorescein isothiocyanate
IELs: Intraepithelial lymphocytes
IL: Interleukin
PBS: Phosphate buffer saline
TNF-α: Tumor necrosis factor-α
ZO-1: Zonula occludens-1
